# G Protein Pathway Suppressor 1 Promotes Influenza Virus Polymerase Activity by Activating the NF-κB Signaling Pathway

**DOI:** 10.1128/mBio.02867-19

**Published:** 2019-12-17

**Authors:** Tomoko Kuwahara, Seiya Yamayoshi, Takeshi Noda, Yoshihiro Kawaoka

**Affiliations:** a Influenza Virus Research Center, National Institute of Infectious Diseases, Tokyo, Japan; b Division of Virology, Department of Microbiology and Immunology, Institute of Medical Science, University of Tokyo, Tokyo, Japan; c Laboratory of Ultrastructural Virology, Institute for Frontier Life and Medical Sciences, Kyoto University, Kyoto, Japan; d Department of Special Pathogens, International Research Center for Infectious Diseases, Institute of Medical Science, University of Tokyo, Tokyo, Japan; e Department of Pathobiological Sciences, School of Veterinary Medicine, University of Wisconsin—Madison, Madison, Wisconsin, USA; St. Jude Children's Research Hospital

**Keywords:** COP9 signalosome, GPS1, NF-kB, influenza, virus-host interactions

## Abstract

Influenza virus relies heavily on cellular machinery to replicate in host cells. Therefore, to better understand the influenza virus life cycle, it is important to identify which host proteins are involved and how they function in virus replication. Previously, we identified G protein pathway suppressor 1 (GPS1) to be a matrix protein 2 (M2)-interacting host protein. GPS1 is a component of the COP9 signalosome, which regulates the NF-κB signaling pathway. Here, we found that the downregulation of GPS1 expression reduced influenza virus replication by more than 2 log units. Although GPS1 was not involved in the early and late stages of virus replication, such as viral entry, uncoating, assembly, or budding, we found that viral polymerase activity was impaired in GPS1-downregulated cells. Moreover, our results suggest that M2 activates the NF-κB signaling pathway in a GPS1-dependent manner and that activation of NF-κB signaling leads to the upregulation of influenza virus polymerase activity. Our findings indicate that GPS1 is involved in the transcription and replication of influenza virus genomic RNA through the activation of the NF-κB signaling pathway.

## Abstract (precis)

In the present study, we identified G protein pathway suppressor 1 (GPS1) to be a host cellular protein that is important for influenza virus replication. We also found that GPS1 plays a role in viral genome transcription through the NF-κB signaling pathway. Moreover, downregulation of GPS1 also affected the growth of vesicular stomatitis virus. Therefore, GPS1 may be a host target for antiviral drugs against influenza virus and possibly other viruses.

## INTRODUCTION

Influenza viruses are obligate intracellular parasites. From entry to budding, viruses rely heavily on their host’s cellular mechanisms to reproduce. Therefore, to control virus replication, it is essential to understand the events that occur in virus-infected cells. In recent years, several large-scale screenings have been performed to identify host factors that could play roles in influenza virus replication (1–9). However, how these host proteins function in the virus replication cycle is not fully understood.

Among influenza virus proteins, matrix protein 2 (M2) is one of the most multifunctional. M2 is a tetrameric integral transmembrane protein (10, 11). The most studied function of M2 is its ion channel activity, which is highly selective for hydrogen ions, in the viral uncoating process (12). Other functions that are also well studied include its roles in the packaging of ribonucleoprotein (RNP) complexes into progeny virions and the budding of progeny virions. When a mutant virus lacking the cytoplasmic tail of M2 is generated by reverse genetics, viral RNPs are not incorporated into the progeny virions, resulting in the release of empty virus particles (13, 14). During progeny virion formation, M2 localizes to the neck of budding virions and alters the membrane curvature, which enables the scission and release of budding virions from the surface of the virus-infected cells (15). In addition, M2 can protect the virus from lysosomal degradation by blocking the fusion of autophagosomes with lysosomes (16). M2 also contributes to the generation of stable progeny virions by binding to LC3, a protein essential for autophagosome formation, and relocalizing LC3 to the plasma membrane of virus-infected cells (17). Recently, it was reported that M2 has an important role in influenza virus activation of inflammasomes by altering the ionic balance of the internal Golgi apparatus (18).

Since M2 works multifunctionally at different subcellular locations and at different virus replication steps, it must interact with many host proteins. Indeed, our group previously identified 207 M2-interacting host proteins that interacted specifically with influenza virus M2 but not with the other influenza virus proteins, by using coimmunoprecipitation and mass spectrometry (8). We noted that all 8 subunits of the COP9 signalosome were included among these M2-interacting host proteins. G protein pathway suppressor 1 (GPS1) is the largest subunit of the COP9 signalosome, a multifunctional protein complex that is necessary for proteasome-dependent protein degradation (19–22). One example of proteasome-dependent protein degradation by the COP9 signalosome is the degradation of the inhibitor of κB (IκB), which forms a complex with NF-κB (23). Degradation of IκB causes the translocation of NF-κB from the cytoplasm to the nucleus, where NF-κB activates numerous transcriptional activities of various host genes, including genes involved in inflammation and the immune response (24). GPS1 localizes to both the nucleus and the cytoplasm and is essential for the maintenance of the COP9 signalosome as a complex (25–27). A recent structural study of GPS1 revealed that its C-terminal tail interacts with IκB, suggesting the possible direct involvement of GPS1 in the regulation of the NF-κB signaling pathway (28).

Because previous studies have suggested that influenza viruses utilize NF-κB for their efficient replication (29–33) and another recent study suggested the possible involvement of the COP9 signalosome in influenza virus replication (34), we selected GPS1, which interacts with M2 and which regulates the NF-κB signaling pathway, for further functional analysis. To better understand the mechanisms of influenza virus replication with respect to virus-host interactions, we attempted to characterize the function of GPS1 in influenza virus replication.

## RESULTS

### GPS1 interacts with M2.

**FIG 1:**
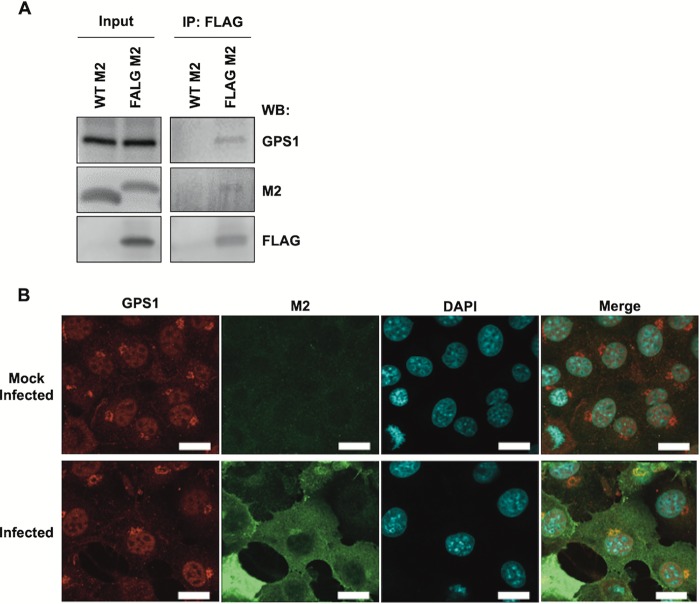
Interaction of GPS1 with M2. (A) The interaction between GPS1 and M2 was assessed by use of a coimmunoprecipitation assay. Either a wild-type (WT) M2- or a FLAG-tagged M2-expressing plasmid was transfected into HEK293 cells. The cells were lysed 24 h after the plasmid transfection and immunoprecipitated with an anti-FLAG monoclonal antibody conjugated with magnetic beads. GPS1 and M2 were detected by Western blotting (WB). IP, immunoprecipitation. (B) The cellular localization of GPS1 and M2 in mock-infected and virus-infected cells was analyzed by using an indirect immunofluorescence assay. Cells were infected with the virus at an MOI of 10 and fixed with 4% paraformaldehyde at 12 h after infection. Endogenous GPS1 was detected by an anti-GPS1 antibody (red), M2 was detected by an anti-M2 antibody (green), and nuclei were stained with Hoechst 33342 (blue). DAPI, 4′,6-diamidino-2-phenylindole. Bars, 20 μm.

To confirm the interaction between M2 and GPS1, we performed an immunoprecipitation assay and Western blotting. Wild-type M2 or FLAG-tagged M2 (which was tagged at its N terminus) was exogenously expressed in 293T cells and subjected to the immunoprecipitation assay. We found that endogenous GPS1 coimmunoprecipitated with the FLAG-tagged M2 but not with wild-type M2, confirming the interaction of GPS1 with M2 (Fig. 1A). Next, to examine the intracellular localization of GPS1, we performed an immunofluorescence assay and observed the localization of GPS1 in virus-infected and mock-infected cells. GPS1 localized to the nucleus, juxtanuclear region, and plasma membrane in mock-infected cells (Fig. 1B), and although no particular change in its subcellular localization in virus-infected cells was observed, GPS1 mainly colocalized with M2 in the juxtanuclear region (Fig. 1B), suggesting that GPS1 interacts with M2 at the juxtanuclear region.

### GPS1 is important for efficient virus replication in cultured cells.

**FIG 2:**
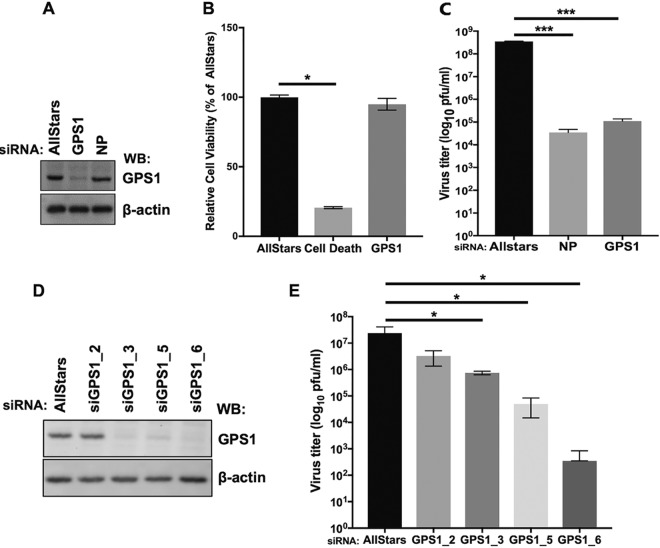
Effect of GPS1 downregulation on virus replication. (A) Downregulation of GPS1 expression by siRNA for GPS1 was examined by Western blotting (WB). (B) A cell viability assay was performed on the siRNA-treated cells. Cell viability was assessed by measuring the amount of ATP in each siRNA-transfected cell. The experiments were performed in triplicate, and the error bars represent the standard deviation for triplicate samples. Statistical analysis was carried out by using analysis of variance (ANOVA), followed by Dunnett’s test. *, *P < *0.05 for three independent experiments. (C) Virus titers in the supernatants of siRNA-transfected cells. The experiments were performed in triplicate, and the error bars represent the standard deviation for triplicate samples. Statistical analysis was carried out by using ANOVA, followed by Dunnett’s test. ***, *P < *0.001 for three independent experiments. (D) The degree of GPS1 downregulation was assessed by using four different siRNAs for GPS1 (siRNA GPS1_2, siRNA GPS1_3, siRNA GPS1_5, and siRNA GPS1_6). GPS1 was detected by Western blotting using the anti-GPS1 antibody. (E) The virus titers in the supernatants of the siRNA-treated cells were examined by using plaque assays at 48 hpi. The experiments were performed in triplicate; the error bars represent the standard deviation for triplicate samples. Statistical analysis was carried out by using ANOVA, followed by Dunnett’s test. *, *P < *0.05 for three independent experiments.

Next, the effect of GPS1 downregulation by small interfering RNA (siRNA) on influenza virus replication was evaluated. GPS1 expression was downregulated by GPS1-targeting siRNA but not by other siRNAs (Fig. 2A); AllStars siRNA (AllStars) has a sequence that is unrelated to that of any mammalian gene and was used as a negative control, whereas siRNA for the influenza virus NP gene was used as a positive control for the reduction in virus growth. To exclude the possibility that GPS1 downregulation was cytotoxic, the viability of the siRNA-transfected cells was examined. The cells were transfected with AllStars siRNA, the siRNA for the GPS1 gene, or the siRNA Cell Death control, which targets cell survival genes, and the amount of ATP in each sample was measured at 48 h after siRNA transfection. The amount of ATP in GPS1-downregulated cells was almost the same as that in the AllStars siRNA-transfected cells, whereas the amount of ATP in the siRNA Cell Death control-transfected cells was about 20% of that in the AllStars siRNA-transfected cells (Fig. 2B). These results indicate that GPS1 downregulation does not affect cell viability. Then, to evaluate the effect of GPS1 downregulation on influenza virus replication, we infected siRNA-treated cells grown in 24-well plates with influenza virus at 500 PFU. At 48 h postinfection (hpi), the supernatants were collected and virus titers were measured by means of plaque assays. The mean virus titer in the supernatant of the AllStars siRNA-transfected cells was 3.6 × 10^8^ PFU/ml, and that in NP-downregulated cells was 3.5 × 10^4^ PFU/ml (Fig. 2C). The virus titer in the supernatant of GPS1-downregulated cells decreased by more than 3 log units compared with that in the supernatant of the AllStars siRNA-transfected cells (Fig. 2C). To confirm the correlation between the degree of GPS1 protein downregulation and the decrease in virus titer, four kinds of siRNAs with different target sequences in the GPS1 gene (siRNA GPS1_2, siRNA GPS1_3, siRNA GPS1_5, and siRNA GPS1_6) were each transfected into HEK293 cells, and then virus infection and titration assessments were performed as in the previous experiments. The results showed that siRNA GPS1_2 did not suppress either GPS1 protein expression (Fig. 2D) or the virus titer (Fig. 2E). The siRNA GPS1_3- and siRNA GPS1_5-transfected cells showed modest degrees of suppression in terms of both GPS1 protein expression and virus titer. The siRNA GPS1_6 produced the greatest degree of suppression of GPS1 protein expression and the greatest decrease in virus titer (Fig. 2D and E). Thus, there was a correlation between the degrees of GPS1 downregulation and the suppression of the virus titer, suggesting that the decrease in virus titer in GPS1-downregulated cells was most likely caused by the downregulation of GPS1.

### GPS1 does not affect the early or late steps of the virus replication cycle.

**FIG 3:**
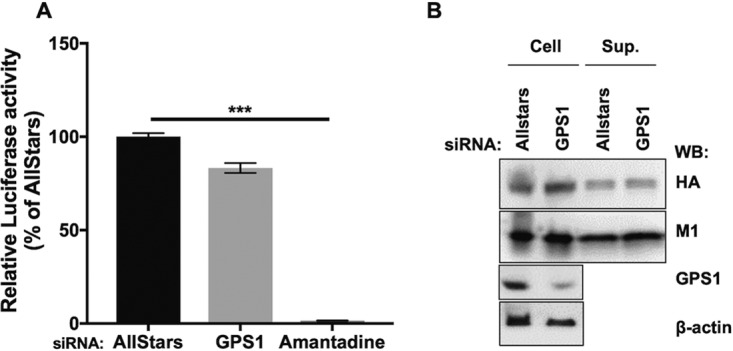
GPS1 does not affect the early or late steps of the virus replication cycle. (A) Effect of GPS1 downregulation on the early steps of influenza virus infection. siRNA-treated cells were infected with PB2-KO/Rluc virus, and luciferase activities in the virus-infected cells were measured at 8 hpi. Amantadine served as a positive control for the inhibition of virus uncoating. Statistical analysis was carried out by using ANOVA, followed by Dunnett’s test. ***, *P < *0.001 for three independent experiments. (B) VLP formation in GPS1-downregulated cells. Viral protein expression plasmids for HA, NA, and M1 were transfected into siRNA-treated cells. The supernatants (Sup.) were harvested at 24 hpi, and HA and M1 were detected by Western blotting using anti-HA and anti-M1 antibodies.

Since M2 is known to be important for the virus uncoating process, we investigated whether GPS1 is involved in the early steps of the virus replication cycle, such as virus entry, uncoating, or the nuclear import of the viral genome, by using a replication-incompetent PB2-knockout virus (the PB2-KO/Rluc virus). The coding region of the PB2 gene of this virus is replaced by the *Renilla* luciferase reporter (Rluc) gene (35). Since this mutant virus lacks a functional PB2 gene, it can go through the steps of host cell attachment, endosomal internalization, uncoating of the viral genome, nuclear import of the viral RNA (vRNA), and initial transcription of vRNA, but it cannot perform *de novo* transcription and replication of the viral genome. At 8 hpi, the virus-infected cells were subjected to the luciferase assay and the levels of luminescence in the virus-infected cells were quantitated. Amantadine, an inhibitor of ion channel activity that inhibits viral uncoating, served as a control. The relative luciferase activity in the amantadine-treated cells was hardly detectable (Fig. 3A). In contrast, the relative luciferase activities were not significantly reduced in the GPS1-downregulated cells compared with those in the AllStars siRNA-transfected cells. These findings suggest that GPS1 is unlikely to be involved in the early steps of influenza virus replication.

Next, we assessed the involvement of GPS1 in the late stages of infection, such as intracellular transport, viral assembly, viral particle formation, and budding, by examining the efficacy of formation of virus-like particles (VLPs), which are induced by the coexpression of the viral proteins hemagglutinin (HA), neuraminidase (NA), and M1 (36). When the expression of HA and M1 in the plasmid-transfected cells was examined, similar levels of HA and M1 proteins were detected in the GPS1-downregulated cells and in the AllStars siRNA-transfected cells. Similar levels of HA and M1 proteins were also detected in the supernatants of GPS1-downregulated cells and the AllStars siRNA-transfected cells (Fig. 3B), suggesting that there were no appreciable differences in the efficiency of VLP formation and release between the negative-control cells and the GPS1-downregulated cells. Therefore, GPS1 is not involved in intracellular transport, viral assembly, viral particle formation, or virus particle budding.

### GPS1 plays a role in the transcription and replication of the virus genome.

**FIG 4:**
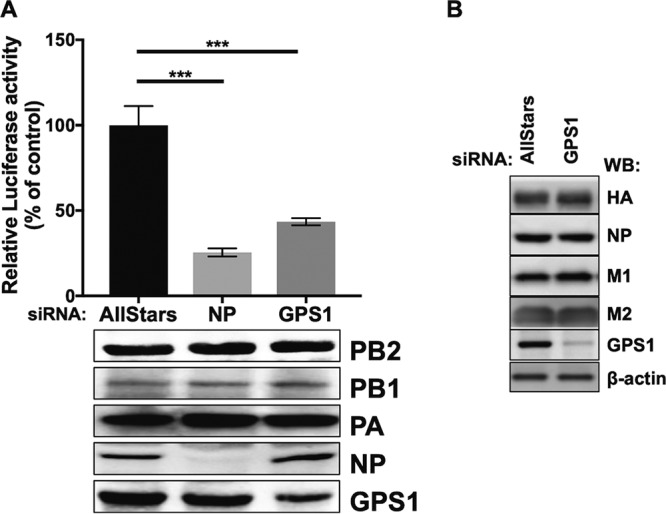
Effect of GPS1 downregulation on influenza virus polymerase activity. (A) Virus polymerase activities in GPS1-downregulated cells were assessed by using a minireplicon assay. The luciferase activity of the AllStars siRNA-transfected cells was set at 100%. The expression of PB2, PB1, PA NP, and GPS1 was examined by Western blotting. The tests were performed in triplicate, and the error bars represent the standard deviation for triplicate samples. Statistical analysis was carried out by using ANOVA, followed by Dunnett’s test. ***, *P < *0.001 for three independent experiments. (B) The stability of the viral proteins was examined. siRNA-treated cells were transfected with HA, NP, M1, or M2 protein-expressing plasmids. The cell lysates were harvested 24 h after the plasmid transfection, and viral proteins were detected by Western blotting.

To determine whether GPS1 plays a role in the transcription and replication of the virus genome, we used a minireplicon assay to compare the viral polymerase activities in GPS1-downregulated cells and AllStars siRNA-transfected cells. In the minireplicon assay, plasmids for the expression of influenza virus polymerase PB2, PB1, PA, and NP, which are necessary for the transcription and replication of vRNA, and a vRNA-expressing plasmid, pPolI NP(0)luc2(0), which carries the firefly luciferase gene between the noncoding regions of the influenza virus NP segment, were cotransfected into siRNA-transfected cells. At 48 h after the plasmid transfection, the cells were subjected to the dual-luciferase assay and the levels of luminescence were quantitated. The luciferase activity in the NP-downregulated cells was 25% of that in the AllStars siRNA-transfected cells. Similarly, the luciferase activity in the GPS1-downregulated cells was almost 40% of that in the AllStars siRNA-transfected cells (Fig. 4A). Because GPS1 is involved in protein degradation via the ubiquitin proteasome system, there was a possibility that the observed decrease in luciferase activity in GPS1-downregulated cells was caused by protein degradation. To eliminate this possibility, we examined the expression levels of the viral proteins in the siRNA-treated cells by transfecting them with polymerase II (Pol II)-dependent protein expression plasmids for the expression of HA, NP, M1, and M2. The cell lysates were harvested 24 h after the plasmid transfection, and the viral proteins were detected by Western blotting. There were no appreciable differences between the expression levels of HA, NP, M1, or M2 in the AllStars siRNA-transfected cells and those in the GPS1-downregulated cells (Fig. 4B). These results indicate that protein expression under the control of the Pol II promoter is not affected by GPS1 downregulation. Therefore, these data suggest that GPS1 is involved in influenza virus polymerase activity.

### Effect of GPS1 downregulation on viral protein and RNA expression.

**FIG 5:**
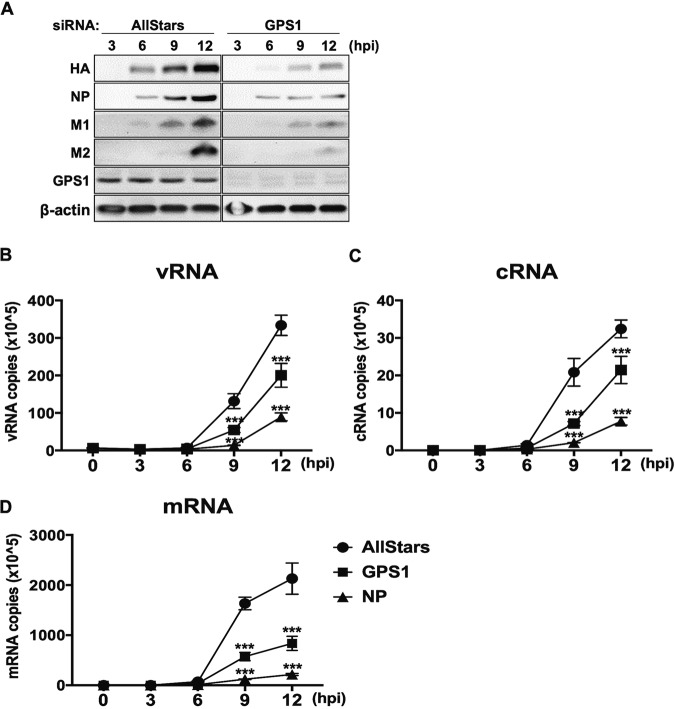
Effect of GPS1 downregulation on influenza virus protein and RNA expression. (A) Effect of GPS1 downregulation on viral protein expression. The expression levels of viral proteins in virus-infected cells were examined. siRNA-treated HEK293 cells were infected with virus at an MOI of 10. The cells were lysed at 3, 6, 9, and 12 hpi, and viral proteins were subjected to Western blotting. (B to D) Effect of GPS1 downregulation on viral RNA expression. The kinetics of the synthesis of the vRNA (B), cRNA (C), and mRNA (D) of the NP segment were examined by quantitative real-time PCR. The experiments were performed in triplicate; error bars represent the standard deviation for triplicate samples. Statistical analysis was carried out by using two-way ANOVA, followed by Sidak’s multiple-comparison test. ***, *P < *0.001 for three independent experiments.

Because the minireplicon assay showed a significant decrease in viral polymerase activity in GPS1-downregulated cells, we next examined the effects of GPS1 downregulation on the expression levels of virus proteins and RNAs in virus-infected cells. First, to confirm that GPS1 plays a role in virus transcription and translation, viral protein expression levels were assessed in the context of the virus infection. Cells transfected with each siRNA were infected with virus at a multiplicity of infection (MOI) of 10, and cell lysates were collected at 3, 6, 9, and 12 hpi. The viral proteins HA, NP, M1, and M2 were then detected by Western blotting. The results showed that viral proteins could be detected from 6 hpi both in AllStars siRNA-transfected cells and in GPS1-downregulated cells but that viral protein expression levels were lower in GPS1-downregulated cells than in AllStars siRNA-transfected cells (Fig. 5A).

To examine whether viral RNA expression levels were also affected by GPS1 downregulation, we quantitated the expression levels of the vRNA, cRNA, and mRNA of the NP segment by using real-time PCR. siRNA-treated cells were infected with virus in the same manner as in the previous experiments, and total RNA was extracted at 0, 3, 6, 9, and 12 hpi. The numbers of RNA copies of all vRNAs, cRNAs, and mRNAs in the NP-downregulated cells were significantly lower than those in the AllStars siRNA-transfected cells at 12 hpi. The numbers of vRNA, cRNA, and mRNA copies in the GPS1-downregulated cells were significantly lower than those in the AllStars siRNA-transfected cells at 9 and 12 hpi (Fig. 5B to D). Therefore, the expression levels of all vRNAs, cRNAs, and mRNAs were impaired in GPS1-downregulated cells, demonstrating that GPS1 is essential for efficient viral transcription and replication.

### GPS1 plays a role in the activation of the NF-κB signaling pathway.

**FIG 6:**
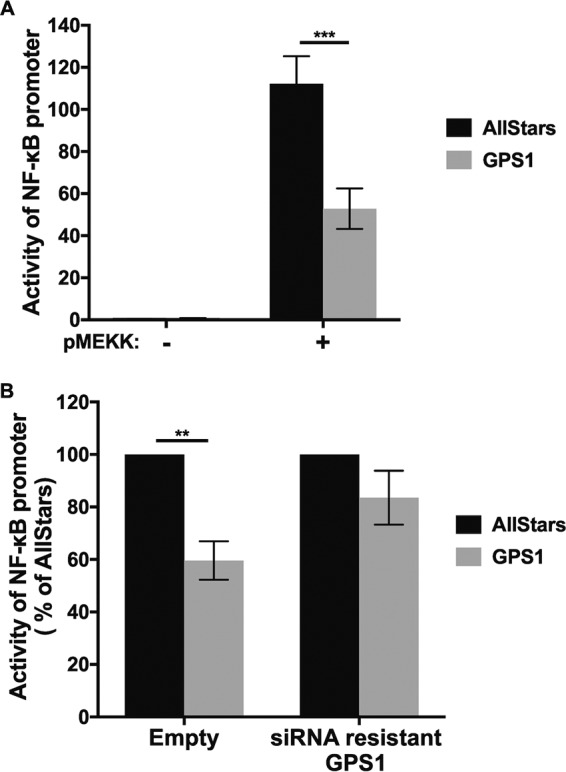
Effect of GPS1 downregulation on NF-κB signaling. (A) The activity of the NF-κB signaling pathway was assessed by using a luciferase assay. HEK293 cells were treated with AllStars siRNA or siRNA for GPS1. Twenty-four hours later, an NF-κB reporter plasmid encoding luciferase and an NF-κB signaling pathway-activating protein (i.e., MEKK)-expressing plasmid were transfected; luciferase activities were measured 24 h after the plasmid transfection. The tests were performed in triplicate, and the error bars represent the standard deviation for triplicate samples. The statistical analysis was carried out by using ANOVA, followed by Sidak's multiple-comparison test. ***, *P < *0.001 for three independent experiments. (B) Recovery of NF-κB signaling pathway activity in GPS1-downregulated cells. Twenty-four hours after siRNA treatment of HEK293 cells, an siRNA-resistant GPS1-expressing plasmid and an NF-κB reporter plasmid encoding luciferase were transfected into the cells. Forty-eight hours later, luciferase activity was measured. The luciferase activity of the AllStars siRNA-transfected cells was set at 100%. The tests were performed in triplicate, and the error bars represent the standard deviation for triplicate samples. Statistical analysis was carried out by using ANOVA, followed by Sidak's multiple-comparison test. **, *P* = 0.002 for three independent experiments.

Because it has been reported that GPS1 interacts with IκB, which negatively regulates NF-κB-dependent transcription (28), and the NF-κB signaling pathway is important for efficient influenza A virus growth (29–33), we investigated the role of GPS1 and the NF-κB signaling pathway in virus replication. To determine whether GPS1 downregulation influences the activation of the NF-κB signaling pathway, we examined the activation of the NF-κB signaling pathway in GPS1-downregulated cells by using a reporter assay. Cells were treated with either AllStars siRNA or siRNA for GPS1. Twenty-four hours later, the pNF-κB-luc plasmid, which encodes the luciferase gene controlled by an NF-κB enhancer element, and a plasmid for the expression of mitogen-activated protein kinase kinase kinase (MEKK), which activates the NF-κB signaling pathway, were cotransfected into the siRNA-treated cells. The cells were lysed after another 24 h, and luciferase activities were measured. Luciferase activity was not detected in either the AllStars siRNA-transfected cells or the GPS1-downregulated cells without MEKK stimulation (Fig. 6A). However, when the AllStars siRNA-transfected cells and the GPS1-downregulated cells were stimulated with MEKK, there was a significant increase in luciferase activities in both cell types. Importantly, the luciferase activity in the GPS1-downregulated cells was significantly lower than that in the AllStars siRNA-transfected cells. These data suggest that GPS1 is important for MEKK-dependent activation of the NF-κB signaling pathway. To confirm that the reduction in NF-κB signaling pathway activity was caused by GPS1 depletion, we performed the reporter assay with exogenously expressed mutant GPS1, which possesses 6 silent mutations in the target site of the GPS1 siRNA. In the cells transfected with the mutant GPS1-expressing plasmid, NF-κB signaling pathway activity was partially restored relative to that in the cells transfected with the empty vector (Fig. 6B). These results suggest that GPS1 does play a role in the NF-κB signaling pathway.

**FIG 7:**
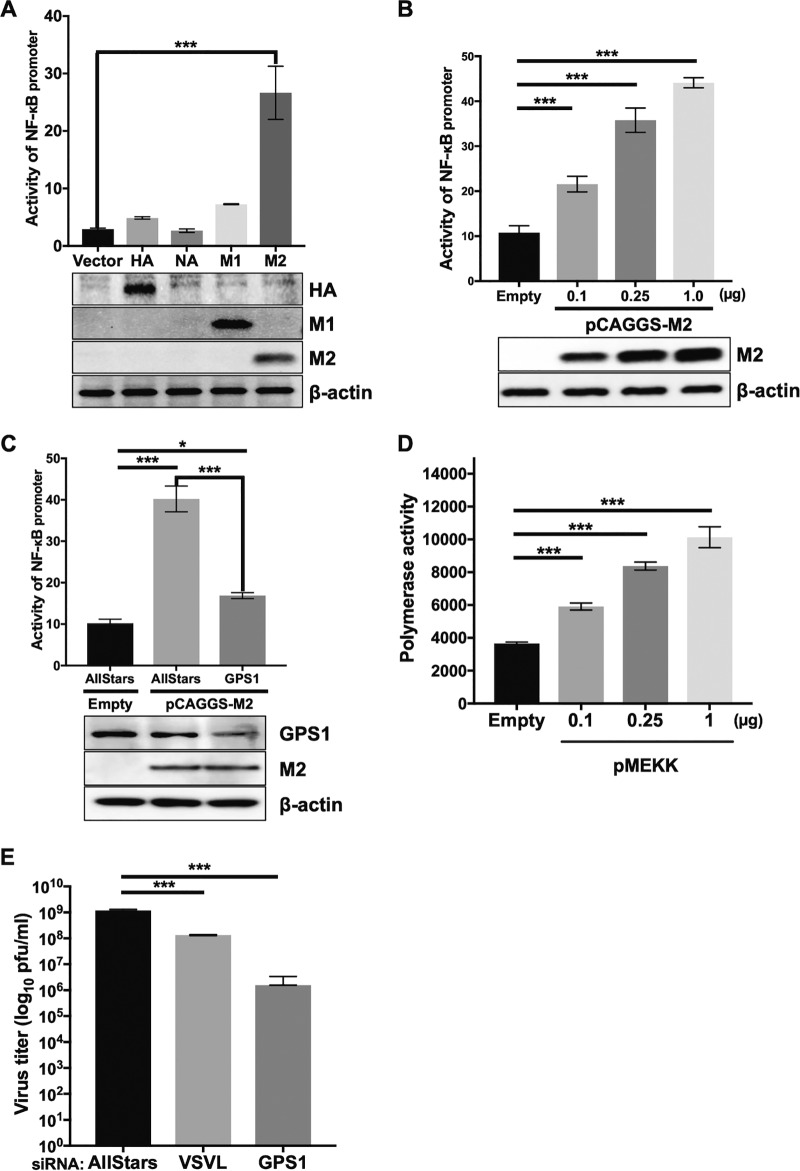
Correlation between GPS1, NF-κB signaling, M2, and influenza virus polymerase activity. (A and B) Activation of NF-κB signaling via the expression of influenza virus proteins. The effect of influenza virus protein expression on NF-κB signaling pathway activation was assessed by expressing the influenza virus M2, M1, HA, or NA protein in cells. Influenza virus M2-, M1-, HA-, or NA-expressing plasmids and an NF-κB reporter plasmid encoding luciferase were transfected into HEK293 cells 24 h after siRNA treatment. Luciferase activities were measured 24 h after the plasmid transfection. The expression of HA, M1, and M2 was examined by Western blotting. The tests were performed in triplicate, and the error bars represent the standard deviation for triplicate samples. Statistical analysis was carried out by using ANOVA, followed by Dunnett’s test. ***, *P < *0.001 for three independent experiments. (C) Effect of GPS1 on the NF-κB signaling pathway when the pathway is activated by M2. HEK293 cells were treated with AllStars siRNA or siRNA for GPS1. Twenty-four hours later, an NF-κB reporter plasmid encoding luciferase and an M2 protein-expressing plasmid were transfected into the cells. Luciferase activities were measured 24 h after the plasmid transfection. The downregulation of GPS1 and the expression of M2 were examined by Western blotting. The tests were performed in triplicate, and the error bars represent the standard deviation for triplicate samples. Statistical analysis was carried out by using ANOVA, followed by Dunnett’s test. *, *P* = 0.013 for three independent experiments; ***, *P < *0.001 for three independent experiments. (D) Virus polymerase activities under the active NF-κB signaling pathway. Polymerase activities were assessed by using a minireplicon assay. An MEKK-expressing plasmid was transfected together with viral polymerase protein-expressing plasmids. Luciferase activities were measured 24 h after the plasmid transfection. The tests were performed in triplicate, and the error bars represent the standard deviation for triplicate samples. The statistical analysis was carried out by using ANOVA, followed by Dunnett’s test. ***, *P < *0.001 for three independent experiments. (E) Effect of GPS1 downregulation in VSV. The titer of VSV in GPS1-downregulated cells was assessed by means of a plaque assay. VSVL, the polymerase L protein of VSV, which is responsible for virus genome transcription and replication. The tests were performed in triplicate, and the error bars represent the standard deviation for triplicate samples. The statistical analysis was carried out by using ANOVA, followed by Dunnett’s test. ***, *P < *0.001 for three independent experiments.

Because GPS1 was originally found as an M2-interacting partner, we then examined whether M2 expression influences the NF-κB signaling pathway. Each protein expression plasmid for M2, M1, HA, and NA was transfected into HEK293 cells with the plasmids required to monitor the activation of the NF-κB signaling pathway. Twenty-four hours after transfection, the cells were lysed and relative luciferase activities were measured. The highest activation level of the NF-κB signaling pathway was seen in the cells transfected with M2 (Fig. 7A). The expression of HA, M1, and M2 was confirmed by Western blotting; NA was not detected because an anti-NA antibody for immunoblotting was unavailable (Fig. 7A). Activation of the NF-κB signaling pathway was observed in an M2 plasmid dose-dependent manner (Fig. 7B). Then, to test whether the M2 expression-induced activation of the NF-κB signaling pathway was dependent on GPS1, the identical reporter assay was performed in GPS1-downregulated cells. NF-κB signaling pathway activity in GPS1-downregulated cell was decreased by more than half of that in AllStars siRNA-transfected cells (Fig. 7C). These results indicate that M2 activates the NF-κB signaling pathway in a GPS1-dependent manner.

Since we found that GPS1 plays a role in both influenza virus polymerase activity and activation of the NF-κB signaling pathway, we attempted to investigate whether activation of NF-κB signaling enhanced viral polymerase activity. The MEKK-expressing plasmid for the activation of the NF-κB signaling pathway was transfected into HEK293 cells with the plasmids for the minireplicon assay. Twenty-four hours after transfection, luciferase activities were measured. The viral polymerase activity increased as the dosage of the MEKK expression plasmid increased (Fig. 7D). Thus, the activation of the NF-κB signaling pathway increased the influenza virus polymerase activity. Taken together, our findings show that M2 activates the NF-κB signaling pathway in a GPS1-dependent manner and that the activation of the NF-κB signaling pathway upregulates influenza virus polymerase activity.

### GPS1 is also important for VSV replication.

As with influenza virus, vesicular stomatitis virus (VSV) is an enveloped virus possessing single-stranded, negative-sense RNA as its genome. Even though VSV replicates in the cytoplasm, a previous study reported that NF-κB signaling is important for the replication of VSV (37). Therefore, we examined the effect of GPS1 downregulation on the growth of VSV. HEK293 cells were treated with AllStars siRNA, siRNA for the GPS1 gene, or siRNA for the VSV polymerase L gene. siRNA-treated cells were infected with VSV at 500 PFU per well of a 24-well tissue culture plate, and the cell supernatants were harvested at 24 hpi. A plaque assay was then performed to determine the virus titer. The virus titer was 1.2 × 10^9^ PFU/ml in the AllStars siRNA-transfected cells (Fig. 7E). The virus titers of the supernatant of the VSV L gene-downregulated cells and of the GPS1-downregulated cells were reduced by about 1 to 3 log units compared with the titer of the supernatant of the AllStars siRNA-transfected cells (Fig. 7E). These data demonstrate that GPS1 also plays an important role in VSV replication.

## DISCUSSION

**FIG 8:**
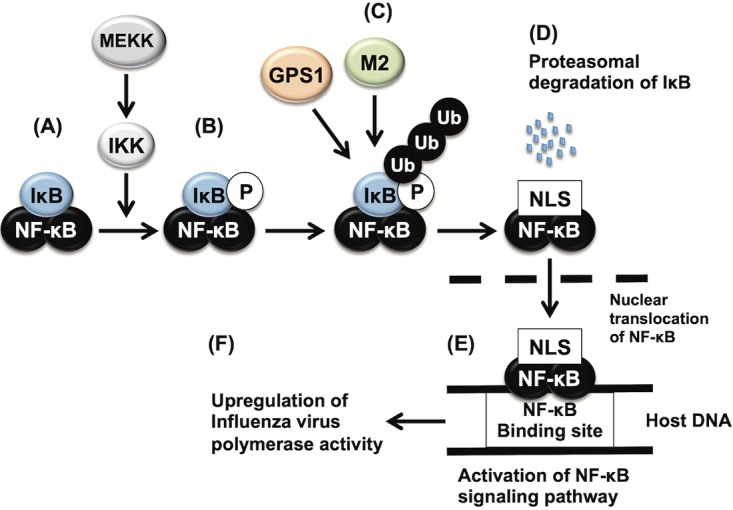
Hypothetical scheme of the role of GPS1 in influenza virus polymerase activity. The results of our study suggest that GPS1 plays a role in influenza virus polymerase activity via the NF-κB signaling pathway. (A) IκB inhibits the nuclear localization of NF-κB by masking the nuclear localization signal of NF-κB. (B) IκB is phosphorylated by IκB kinase (IKK). (C) GPS1, possibly with M2, supports the polyubiquitination of IκB. (D) Polyubiquitinated IκB is recognized by proteasomes and subjected to proteasomal degradation, and the NLS of NF-κB is exposed. (E) NF-κB translocates to the nucleus, and the NF-κB signaling pathway is activated. (F) Finally, the activation of the NF-κB signaling pathway results in the upregulation of influenza virus polymerase activity. Ub, ubiquitin.

NF-κB signaling is required for the efficient replication of influenza virus (29–33). Here we demonstrated that the downregulation of GPS1 severely affects viral polymerase activity (Fig. 4A) and NF-κB signaling activity (Fig. 6A), indicating that GPS1 supports both of these activities. However, the role of GPS1 in viral polymerase activity is thought to be indirect since GPS1-dependent NF-κB activation via MEKK expression also enhanced virus polymerase activity (Fig. 7D). Because the results of our study suggested that GPS1 plays a role in influenza virus polymerase activity via the NF-κB signaling pathway, we illustrated a possible mechanism (Fig. 8A to F): MEKK and IκB kinase (IKK) are activated upon virus infection (38) (Fig. 8A), IκB is phosphorylated by IKK (39) (Fig. 8B), GPS1 and M2 support the polyubiquitination of IκB (Fig. 8C), polyubiquitinated IκB is recognized by proteasomes and subjected to proteasomal degradation, the nuclear localization signal (NLS) of NF-κB is exposed (40, 41) (Fig. 8D), NF-κB translocates to the nucleus, and the NF-κB signaling pathway is activated (42) (Fig. 8E); finally, activation of the NF-κB signaling pathway results in the upregulation of influenza virus polymerase activity (Fig. 8F). However, when we examined the cellular localization of NF-κB, it did not clump together at the juxtanuclear region like GPS1 and M2. Further studies are required to elucidate the detailed mechanism of how all these proteins work together.

We also found that M2 expression activated the NF-κB signaling pathway in a GPS1-dependent manner (Fig. 7A to 7C). However, just as the NF-κB signaling pathway was activated without M2 expression (Fig. 6A), M2 would be one of many NF-κB signaling pathway activators. Thus, it seems that the NF-κB signaling pathway activation induced by virus infection occurs as a result of a compound effect of multiple activators of the pathway. Further investigation is required to elucidate the detailed mechanisms by which M2 activates the NF-κB signaling pathway.

In this study, our results showed that not only the replication of influenza virus but also that of VSV was suppressed in GPS1-downregulated cells (Fig. 7E). This result is consistent with that of a previous study by Shulak et al. (37). Many viruses, such as human immunodeficiency virus type 1 (HIV-1), cytomegalovirus, herpesvirus, human papillomavirus 16, hepatitis B virus, and Epstein-Barr virus (EBV), utilize the NF-κB signaling pathway in their replication cycles (43). It is possible for GPS1 to play a role in the replication of all of these viruses. Therefore, a compound that can control the function of GPS1 could become a universal antidote against viruses that depend on NF-κB signaling for their growth.

In conclusion, we demonstrated that the host protein GPS1 contributes to influenza virus replication by supporting the transcription and replication of the influenza virus genome through NF-κB signaling. Since GPS1 also contributes to VSV replication, this host protein may be used by many negative-strand RNA viruses. Further unveiling of the functions of GPS1 may lead to the discovery of an antiviral drug that is effective against a variety of viral infections.

## MATERIALS AND METHODS

### Cells and viruses.

Human embryonic kidney (HEK293) cells were purchased from ATCC and were cultured in Dulbecco’s modified Eagle medium (Sigma-Aldrich, St. Louis, MO) supplemented with 10% heat-inactivated fetal calf serum (FCS) and a penicillin-streptomycin solution (Sigma-Aldrich, St. Louis, MO). Madin-Darby canine kidney (MDCK) cells were obtained from Robert Webster (St. Jude Children’s Research Hospital, Memphis, TN) and were grown in Eagle’s minimal essential medium supplemented with 5% newborn calf serum (NCS). Baby hamster kidney (BHK) cells were obtained from Ayato Takada (Hokkaido University, Japan) and were cultured in Dulbecco’s modified Eagle medium containing 5% FCS. The cells were incubated at 37°C in 5% CO_2_. Influenza A/WSN/33 (WSN) virus (H1N1) was generated by use of reverse genetics as described previously (44) and propagated in MDCK cells. The vesicular stomatitis virus (VSV) Indiana strain was kindly provided by Michael Whitt (University of Tennessee) and was propagated in BHK cells.

### Plasmids and transfection reagents.

Plasmids for the expression of GPS1 were constructed by using RNA extracted from HEK293 cells as a template for reverse transcription-PCR (RT-PCR). The PCR product was inserted into pCAGGS/MCS (45). Viral RNA-expressing plasmids or viral protein-expressing plasmids were generated as described elsewhere (44). pNF-κB-luc and pMEKK were purchased from Stratagene (Stratagene, La Jolla, CA). Plasmids were transfected into HEK293 cells with the TransIT-293 reagent according to the manufacturer’s protocol (Mirus, Wisconsin, WI).

### Antibodies.

Rabbit anti-GPS1 (clone 16-30), mouse anti-β-actin (clone AC-74), mouse anti-FLAG epitope (clone M2), and anti-FLAG M2 magnetic beads were purchased from Sigma-Aldrich (St. Louis, MO). Mouse anti-M2 antibody (antibody rM2ss23) (46), anti-PB2 antibody (antibody 18/1) (47), anti-PB1 antibody (antibody 136/1), and anti-PA antibody (antibody 55/2) (48) were kindly provided by Ayato Takada, Hokkaido University. The mouse anti-M1 antibody (antibody WS-27/52), mouse anti-HA antibody (antibody WS3-54), and mouse anti-Aichi NP antibody (antibody 2S-347/3) were produced in our laboratory at the University of Wisconsin.

### Western blotting.

Protein samples were lysed with 1× Tris-glycine SDS sample buffer (Invitrogen, Carlsbad, CA) containing 10 mM dithiothreitol. Then, the lysates were treated at 95°C for 10 min and immediately placed on ice. When the samples were prepared, each sample was applied to an Any kD Mini-Protean TGX gradient gel (Bio-Rad, Hercules, CA). The proteins in the gel were transferred electrophoretically to polyvinylidene difluoride membranes (Millipore, Bedford, MA) with transfer buffer (100 mM Tris, 190 mM glycine, 10% methanol). The membranes were blocked by using the Blocking One reagent (Nacalai Tesque, Kyoto, Japan), and the signals were detected by using a Chemi-Lumi One Super assay kit (Nacalai Tesque, Kyoto, Japan) and visualized with a VersaDoc imaging system (Bio-Rad, Hercules, CA).

### Indirect immunofluorescence assay.

HEK293 cells were infected with influenza virus at a multiplicity of infection (MOI) of 10. Cells were fixed with 4% paraformaldehyde (PFA) and permeabilized with 0.2% Triton X-100. A confocal microscope with an LSM510 system (Carl Zeiss, Oberkochen, Germany) was used for microscopic examinations. Nuclei were stained with Hoechst 33342 (Invitrogen, Carlsbad, CA).

### Immunoprecipitation assay.

FLAG-tagged M2 and M2 protein-expressing plasmids were each transfected into HEK293 cells by using the TransIT-293 transfection reagent. Twenty-four hours later, the cells were washed with phosphate-buffered saline and lysed with 1 ml of lysis buffer (50 mM Tri HCl [pH 7.5], 150 mM NaCl, 1 mM EDTA, 0.5% Nonidet P-40, protease inhibitor mixture [cOmplete mini; Roche]) for 1 h at 4°C. Then, the cellular debris in each sample was removed by centrifugation. The supernatants were then incubated with 10 μl of anti-FLAG M2 magnetic beads (Sigma-Aldrich, St. Louis, MO) overnight at 4°C. After the affinity gels were washed three times with lysis buffer, 50 μl of 1× sample buffer was added to the samples. Finally, the samples were subjected to SDS-PAGE followed by Western blotting.

### siRNA treatment.

siRNAs for GPS1 were purchased from Qiagen as part of a predesigned genome-wide human siRNA library (FlexiTube siRNA; Qiagen). The following siRNA target sequences for GPS1 were used: GPS1_2 (AAG AGC AGA CTC AGC GTT AAA), GPS1_3 (CAA GTG GGC GGT GTC CAT TAA), GPS1_5 (AAC CTT TAA CGT GGA CAT GTA), and GPS1_6 (CAG CCT GGA TCT GGA ACA GTA). siRNA GPS1_6 was used for all GPS1 downregulation experiments. AllStars siRNA (Qiagen, Tokyo, Japan) was used as a negative control. The siRNA targeting the WSN virus NP gene (GGA UCU UAU UUC UUC GGA GUU) was purchased from Sigma-Aldrich. HEK293 cells, cultivated in 24-well plates, were transfected with 25 nM the indicated siRNA by using the Lipofectamine RNAiMAX reagent (Invitrogen, Carlsbad, CA). Downregulation of the targeted genes was evaluated by the use of Western blotting.

### Virus titration.

To assess influenza virus replication, two parallel sets of siRNA-transfected cells were infected with WSN virus at 500 PFU per well of a 24-well tissue culture plate at 24 h after the second siRNA transfection. At 48 h postinfection, the supernatants were harvested and the virus titers were determined by means of plaque assays in MDCK cells.

### Minireplicon assays.

Following siRNA treatment, influenza virus RNA polymerase activity was assessed by using a minireplicon assay as described previously (49). To investigate influenza virus polymerase activity when the NF-κB signaling pathway was activated, mitogen-activated protein kinase kinase kinase (MEKK) was additionally expressed by means of plasmid transfection. A plasmid encoding *Renilla* luciferase was used as the transfection control.

### Cell viability assays.

To evaluate the viability of siRNA-treated cells, cell lysates were collected after 48 h of siRNA transfection, and viability was measured by using a CellTiter-Glo assay system (Promega, Madison, WI) according to the manufacturer’s instructions.

### Quantitative reverse transcription-PCR.

siRNAs for the negative control, the influenza WSN NP gene, and GPS1 were transfected into HEK293 cells as described above. siRNA-transfected cells were then infected with WSN virus at an MOI of 10 at 37°C for 1 h, and cell lysates were collected at 0, 3, 6, 9, and 12 h postinfection. Total RNA was extracted by using a Maxwell 16 LEV simplyRNA tissue kit (Promega, Madison, WI). Reverse transcription and quantitation of vRNA, cRNA, and mRNA were performed by real-time RT-PCR as previously described (50).

### PB2-KO/Rluc virus assay.

AllStars siRNA or siRNA for GPS1 was transfected into HEK293 cells as described above. A replication-incompetent PB2-knockout virus (PB2-KO/Rluc virus) (35), which possesses the *Renilla* luciferase reporter gene in the coding region of the PB2 segment, was used to infect siRNA-transfected cells at an MOI of 1. At 8 h postinfection, the cells were lysed and the relative luciferase activities were measured by using a *Renilla* luciferase assay system (Promega, Madison, WI). The ion channel inhibitor amantadine (100 μg/ml) was used as a control.　

### Virus-like particle (VLP) formation assay.

HEK293 cells were treated with the negative-control AllStars siRNA and siRNA targeting the GPS1 gene as described above. siRNA-treated HEK293 cells were then transfected with HA-, NA-, and M1-expressing plasmids by using the TransIT-293 reagent (Mirus, Madison, WI) according to the manufacturer’s instructions. Forty-eight hours after the plasmid transfections, the cell lysates and supernatants were collected. The supernatants were centrifuged at 3,000 × *g* for 5 min at 4°C to remove the cell debris. Then, the supernatants were layered over a 20% sucrose cushion, concentrated by ultracentrifugation at 50,000 rpm for 2 h at 4°C, and pelleted. The cell lysates and the pellets were then analyzed by SDS-PAGE and Western blotting.

### NF-κB signaling pathway reporter assay.

HEK293 cells were treated with the negative-control AllStars siRNA and siRNA targeting the GPS1 gene. The siRNA-treated cells were then transfected with 250 ng of pNF-κB-Luc and 25 ng of pMEKK. Twenty-four hours later, the cells were lysed and the levels of luciferase activity were determined by using a dual-luciferase reporter assay system (Promega, Madison, WI). A plasmid encoding *Renilla* luciferase was used as the transfection control.

To investigate whether viral proteins could activate the NF-κB signaling pathway, 1 μg of M2, M1, HA, or NA protein-expressing plasmid was transfected into the HEK293 cells instead of the MEKK-expressing plasmid. The luciferase activities were then measured at 24 h posttransfection.

M2-activated NF-κB signaling pathway activity in GPS1-downregulated cells was evaluated by transfecting the cells with the M2 protein-expressing plasmid and reporter plasmids 24 h after siRNA treatment. Twenty-four hours after the plasmid transfection, luciferase activities were measured.
